# Microorganisms involved in deep neck infection (DNIs) in Greece: detection, identification and susceptibility to antimicrobials

**DOI:** 10.1186/s12879-019-4476-3

**Published:** 2019-10-15

**Authors:** Despoina Beka, Vasileios A. Lachanas, Stergios Doumas, Stelios Xytsas, Anastasios Kanatas, Efi Petinaki, Charalampos Skoulakis

**Affiliations:** 1 Department of Otorhinolaryngology, University Hospital of Larissa, Larissa, Greece; 2 OMFS Department, Leeds Teaching Hospitals and St James Institute of Oncology, Leeds Dental Institute and Leeds General Infirmary, Leeds, UK; 3 Department of Microbiology, Medical School, University of Thessaly, Larissa, Greece

**Keywords:** Neck abscesses, Neck infection, Neck spaces, Causative agents, Epidemiology

## Abstract

**Background:**

To determine, from October 2010 to October 2018, the epidemiology of Deep Neck Infections (DNIs), regarding the detection, the identification and the susceptibility to antimicrobials of causative microorganisms, in Thessaly-Central Greece.

**Methods:**

An analysis of data from a prospective database was conducted on 610 consecutive patients with DNIs treated in the Otolaryngology / Head & Neck Surgery Department of University Hospital of Larissa. Demographics, clinical features and microbiological data were analyzed.

**Results:**

Among the 610 patients (1,9/1 male to female ratio, mean age: 39,24 ± 17,25) with DNIs, 579 had a single space (94,9%), while the remaining 31 had a multi-space (5,1%) DNI. The most common areas affected were the peritonsillar space (84,6%) followed by the submandibular space (6,5%). Clinical samples were obtained from 462 patients, and were tested by culture and by the application of 16S rRNA PCR. Two hundred fifty-five samples (55,2%) gave positive cultures, in which *Streptococcus pyogenes* and *Staphylococcus aureus* were predominant. The application of the 16S rRNA PCR revealed that 183 samples (39,6%) were positive for bacterial DNA; 22 of them, culture negative, were found to be positive for anaerobic (*Fusobacterium necrophorum, Actinomyces israellii* etc) and for fastidious microorganisms (*Brucella mellitensis, Mycobacterium avium*).

**Conclusion:**

DNIs represent a medical and surgical emergency and evidence-guided empirical treatment with intravenous infusion of antibiotics at the time of diagnosis is mandatory, highlighting the importance of epidemiological studies regarding the causative microorganisms. Although, in our study, the predominant pathogens were *S. pyogenes* and *S. aureus,* the combination of culture and molecular assay revealed that anaerobic bacteria play also a significant role in the pathogenesis of DNIs. Based on the local epidemiology, we propose as empirical therapy the intravenous use of a beta-lactam /beta-lactamase inhibitor; metronidazole or clindamycin can be added only in specific cases such as in immunocompromised patients.

## Background

Deep neck infections (DNIs) are defined by the presence of inflammation with or without pus in the deep spaces and fasciae of the head and neck. DNIs can be categorized into parapharyngeal, infratemporal, pterygomaxillary, temporal, parotid, masticator, submandibular, visceral, carotid sheath, peritonsillar-pharyngeal mucosal, retropharyngeal, danger and prevertebral spaces [1–3]. Despite the improvements of diagnostic tools (imaging and microbiological techniques), DNIs continue to be fatal, especially in immunocompromised patients or patients with significant co-morbidities [4]. Their severity and extent can be overlooked, often masquerading other infections (i.e. pharyngitis, tonsillitis, torticollis etc), thus leading to delayed diagnosis [5].

Adult DNIs more commonly involve multiple spaces, leading to severe complications and appear to be more serious compared to children [6]. In addition, use of analgesic, anti-inflammatory drugs and corticosteroids may mask presentations by blunting immune responses. It is sometimes difficult to trace the origin of the infection, since the primary source of infection may precede by weeks, given that the clinical manifestations are diverse and depend on the affected spaces [7]. Even today, DNIs continue to be fatal, leading to life-threatening complications such as airway compromise, pneumonia, pericarditis, jugular vein thrombosis, mediastinal involvement. Treatment principles consist of adequate resuscitation, with surgical drainage of the neck and management of complications, combined with appropriate antimicrobial therapy. Although it is better to obtain cultures before the antibiotic treatment, the patients often are empirically treated, according to local and international guidelines.

In Greece, antimicrobial agents used for the treatment of DNIs include the intravenous infusion, alone or in combination, of penicillin, amoxicillin plus clavulanic acid, ampicillin plus sulbactam, clindamycin and metronidazole. However, the emergence and the spread of multi-drug bacteria both in the nosocomial environment and in the community emphasizes the need for a large epidemiological survey focused on the etiology and on the susceptibility of microorganisms that are the causative agents of DNIs.

The purpose of this study was to determine, during an eight- years study period (October 2010–October 2018) the identification and the susceptibility to antimicrobials of microorganisms involved in DNIs in Central Greece, in order to avoid clinical failure and misuse of antibiotics.

## Methods

### Patients with DNIs

A study of data exported from a prospective database was conducted on 610 consecutive patients with DNI diagnosis, admitted from October 2010 until October 2018 in the Otolaryngology / Head & Neck Surgery Department of University Hospital of Larissa, which is the main tertiary hospital of Thessaly, Central Greece. Thessaly is a rural area of Greece with about 1.000.000 inhabitants. The diagnosis of deep neck abscess was suspected by clinical history and confirmed by Computed Tomography (CT) or surgery. Demographic data (name, age, gender, residence, occupation, travel, previous hospitalization) and clinical information (underlying disease, antibiotic therapy) of the patients were collected. Clinical samples were obtained, after admission and before starting antibiotic treatment, by needle aspiration or by sterile swab using the BD ESwab™ collection and transport system (Becton Dickinson) and were immediately sent to the Microbiological Laboratory. The purulent material was divided in two parts, one for Gram-stain and culture and one for molecular assay.

### Microbiological methods

#### Conventional methods: gram-stain, culture, identification and antimicrobial susceptibility test

The specimens, after being tested by Gram-stain, were cultured on two blood agar plates (one aerobically and one anaerobically using BD BBL™ GasPak™ anaerobic), on Mc Conkey agar and on Sabouraud agar for 5 days at 37 °C. The identification of microorganisms to the species level and the susceptibility testing was performed by the VITEK®2 automated system (bioMérieux, Marcy-l’Étoile, France). MICs were interpreted according to the European Committee on Antimicrobial Susceptibility Testing (EUCAST) (http://www.eucast.org/clinical_breakpoints/).

#### Molecular methods: 16S rRNA PCR followed by sequencing analysis

In an effort to identify rapidly the causative agent of DNIs, application of the 16S rRNA PCR was performed directly to the specimens as previously reported [9]. Briefly, DNA was extracted using a QI Amp DNA Mini kit (QIAGEN, Hilden, Germany), according to the instructions of the manufacturer. Then, the *16S rRNA* gene was amplified using the universal primers 5΄-AGAGTTTGATCATGGCTCA-3΄ (forward; located at positions 8 to 27) and 5΄-ACGGCGACTGCTGCTGGCAC-3΄ (reverse; positions 531 to 514 *Escherichia coli*). In the case where a band of approximately 520 bp was obtained, PCR amplicons were sequenced in both directions in an ABI 3130 genetic analyzer and were compared with those submitted to GenBank and EMBL, using the BLAST algorithm.

### Statistical analysis

All descriptive data were reported in percentages. 2 × 2 contingency tables and Chi-Square tests were used to assess potential correlations. Data analysis was performed with the SPSS 20 statistical software (IBM, Chicago, IL, USA) and values of *p* < 0.05 were considered as significant results.

## Results

Among the 610 patients with DNIs, 399 were male and 211 females (1,9/1 male to female ratio) with a mean age ± SD of 39,24 ± 17,25 years. Among them, 564 were adults (≥ 18 years old) and 46 children (< 18 years old). In adults single space involvement was noticed in 534 patients (94,7%), and multi-space involvement in 30 patients (5,3%) (in 14 patients DNIs were located in more than two neck spaces). Peritonsillar- pharyngeal mucosal was the most common space involved (88,3%) followed by submandibular space (7,2%). In children single space involvement was noticed in 45 patients (97,8%) and multi-space involvement in one patient (2,2%). Peritonsillar was the most common space involved (86,9%) followed by parapharyngeal space (6,5%) (Table 1).

**Table 1:** Demographics and spaces involved of the 610 patients with deep neck infection

All patients (*n* = 610)	male/female: 399/211	mean age ± SD: 39,24 ± 17,25
Space involved	Single space (*n* = 579)	Multi-space (*n* = 31)
- Peritonsillar-PMS^a^	516	5
- Submandibular	40	17
- Para-pharyngeal	10	20
- Retropharyngeal	7	6
- Masticator	3	6
- Parotid	3	
- Visceral		6
- Ludwig’s angina		5
- Danger		5
- Carotid		1
Adults (*n* = 564)	male/female: 378/186	mean age ± SD: 41,39 ± 16,06
Space involved	Single space (*n* = 534)	Multi-space (*n* = 30)
- Peritonsillar-PMS	476	4
- Submandibular	39	16
- Para-pharyngeal	7	20
- Retropharyngeal	6	6
- Masticator	3	6
- Parotid	3	
- Visceral		6
- Ludwig’s angina		5
- Danger		5
- Carotid		1
Children (*n* = 46)	male/female: 21/25	mean age ± SD: 12,8 ± 4,68
Space involved	Single space (*n* = 45)	Multi-space (*n* = 1)
- Peritonsillar-PMS	40	1
- Para-pharyngeal	3	1
- Retropharyngeal	1	
- Submandibular	1	

^a^*PMS* pharyngeal mucosal space

All patients underwent either needle aspiration or surgical drainage; cultures were taken before the empirical antimicrobial therapy was started. In some cases the treatment was modified properly after the results of susceptibility testing. Specimens for microbiological analysis were obtained in 462 out of 610 patients (428 adults and 34 children), while 45,6% (210/462) of them (46,3% of adults and 35,3% of children) had taken antibiotics before admission (Table 2). The most frequently antibiotics used before admission were amoxicillin plus clavulanic acid and clarithromycin (peros); empirical therapy included intravenous infusion of ampicillin-sulbactam combined with metronidazole or clindamycin. We note that clinical samples for microbiological analysis were not taken from 148 patients (24%), due mainly to the insufficient purulent material.

**Table 2:** Data of prior antibiotic therapy and culture results for all our patients (children and adults)

	Prior antibiotic therapy		
	No	Yes	Total
Culture results			
Negative	112	95	207
Positive	140	115	255
Total	252	210	462

From the 462 clinical specimens, 55,2% (255/462) yielded positive cultures, while in 3 clinical samples two different bacterial species were isolated; thus 258 bacterial species were totally collected (see Tables 3 and 4). Chi-Square tests did not demonstrate any significant difference (*p* > 0,05) between prior antibiotics uptake and positive or negative culture results (Table 5), maybe due to insufficient power. From the isolated bacteria species 91,9% (237/258) were aerobic and 8,1% (21/258) anaerobic. The most common aerobic bacteria were *Streptococcus pyogenes* (45,3%) and *Staphylococcus aureus* (26,7%). The most common anaerobic bacteria were *Prevotella melaninogenica* (2,7%) and *Fusobacterium nucleatum* (2,7%) (Table 3). No significant correlation was noted in culture positivity from adults vs. children (Pearson Chi-Square: 2301; P: 0,129 > 0.05 / Yate’s Continuity Correction: 1790; P: 0,181 > 0.05 / Fisher’s Exact Test: P: 0,153 > 0.05).

**Table 3:** Distribution of the species isolated from the positive cultures of adult (*n* = 232) and children (*n* = 23) with DNIs patients. Three cultures were positive for two microorganisms

	Adults (*n* = 232)		Children (*n* = 23)	
	No	(%)	No	(%)
Aerobic Bacteria	215	92,7	22	95,7
a. Gram-positive	207	89,2	19	82.6
*1. Streptococcus pyogenes*	*104*	*44,8*	*13*	*56,5*
*2. Staphylococcus aureus*	*65*	*28*	*4*	*17,4*
*3. Streptococcus group C*	*23*	*9,9*	*1*	*4,3*
*4. Streptococcus anginosus*	*11*	*4,7*	*1*	*4,3*
*5. Streptococcus constellatus subsp. pharynges*	*2*	*0,9*		
*6. Streptococcus sanguinis*	*1*	*0,4*		
*7. Staphylococcus epidermidis*	*1*	*0,4*		
b. Gram-negative	8	3,4	3	13
*1. Pseudomonas aeruginosa*	*3*	*1,3*		
*2. Klebsiella pneumoniae*	*2*	*0,9*	*1*	*4,3*
*3. Proteus mirabilis\*	*2*	*0,9*		
*4. Serratia liquefaciens*	*1*	*0,4*		
*5. Providencia stuartii*			*2*	*8,7*
Anaerobic Bacteria	20	8,6	1	4,3
a. Gram-positive	6	2,6	1	4,3
*1. Parvimonas micra*	*5*	*2,2*		
*2. Clostridium bifermentans*	*1*	*0,4*	*1*	4,3
b. Gram-negative	14	6		
*1. Prevotella melaninogenica*	*7*	*3*		
*2. Fusobacterium nucleatum*	*7*	*3*

**Table 4:** The results of Chi-Square tests did not demonstrate any significant difference (*p* > 0,05) between prior antibiotics uptake and positive or negative culture results

	Value	P Asymptotic Significance (2-sided)	P Exact Significance (2-sided)	P Exact Significance (1-sided)
Pearson Chi-Square	0,029	0,864		
Yate’s Continuity Correction	0,006	0,939		
Likelihood Ratio	0,029	0,864		
Fisher’s Exact Test			0,925	0,469

**Table 5:** Detection of the bacterial species involved in DNIs of adults and children (in 3 cultures two different bacteria species were isolated)

	Culture	Culture + 16SrRNA	16SrRNA
Aerobic Bacteria	237	143	3^a^
a. Gram-positive	226	137	
*1. Streptococcus pyogenes*	*117*	*69*	
*2. Staphylococcus aureus* (2 MRSA)	*69*	*50*	
*3. Streptococcus group C*	*24*	*13*	
*4. Streptococcus anginosus*	*12*	*5*	
*5. Streptococcus constellatus subsp. pharynges*	*2*		
*6. Streptococcus sanguinis*	*1*		
*7. Staphylococcus epidermidis*	*1*		
*8. Brucella melitensis*			2^a^
*9. Mycobacteriun avium*			1^a^
b. Gram-negative	11	6	–
*1. Klebsiella pneumoniae*	*3*	*3*	
*2. Pseudomonas aeruginosa*	*3*	*3*	
*3. Providencia stuartii*	*2*		
*4. Proteus mirabilis*	*2*		
*5. Serratia liquefaciens*	*1*		
Anaerobic Bacteria	21	7	19^a^
a. Gram-positive	7	*0*	11^a^
*1. Parvimonas micra*	*5*		
*2. Clostridium bifermentans*	*2*		
*3. Actinomyces israelii*			11^a^
b. Gram-negative	14	*7*	8^a^
*1. Prevotella melaninogenica*	*7*	*4*	
*2. Fusobacterium nucleatum*	*7*	*3*	
*3. Fusobacterium necrophorim*			8^a^

^a^Results obtained only by 16S rRNA PCR

Regarding the susceptibility of aerobic Gram-positive cocci to beta-lactams, all streptococci as anticipated were susceptible to penicillin. Among staphylococci, 90% of *S. aureus* isolates were resistant to penicillin and 3,9% were methicillin-resistant (MRSA). High rate of resistance to macrolides and lincosamides were observed in both *S. pyogenes* and *S. aureus* isolates (17 and 19% respectively). Aerobic Gram-negative bacteria expressed a wild-type phenotype without additional acquired resistance mechanisms. Finally, all anaerobic bacteria which were isolated by culture, were susceptible to amoxicillin-clavulanic, ampicillin-sulbactam, clindamycin and metronidazole.

The application of 16S rRNA PCR directly to the specimens revealed the presence of bacterial DNA in 183 out 462 samples (39,6%). One hundred-fifty of them (82%) gave positive cultures; identification by sequencing analysis was in concordance with that obtained by conventional methods. Results obtained by this molecular method were available within 2 days after the sampling. In addition, among the 33 samples (18%), that were PCR positive but culture negative, 22 (12%) were found to be positive for DNA of fastidious microorganisms, such as *Actinomyces israelii* (11 out 22), *F. necrophorum* (8 out 22), *Brucella melitensis* (2 out 22) and *Mycobacterium avium* (1 out 22). Regarding the remaining 11 samples (6%), Sanger sequencing analysis failed to distinguish the microorganisms involved, given that a polymicrobial genetic pattern was obtained.

## Discussion

DNIs are potentially fatal and require an aggressive diagnostic and therapeutic management. In the pre-antibiotic era, pharyngeal/tonsillar infection were responsible for 70% of deep neck space infection [10, 11]. Usually, DNIs occur after previous uncontrolled infections such as tonsillitis, dental infections, surgery, head and neck trauma or lymphadenitis after upper airways infection [4, 12], while, it is sometimes difficult to find the origin of DNI because the primary source of infection may precede it by weeks [7].

The management of DNIs involve surgical or needle drainage of the abscess associated with the use of intravenous antibiotics [13–15]. DNIs require timely treatment with IV antibiotics at the time of diagnosis because of the rapidly progressive nature of these infections. Antibiotic therapy should be empirically initiated, based on local epidemiology, ideally before culture and sensitivity results are available [1]. Until now, various empiric antibiotics for deep neck infection have been proposed [16–21]. This fact highlights the importance of epidemiological studies in DNIs microbiology, since these studies help to determine the proper empirical treatment in each geographical area. To our knowledge, this is the first study in Greece focused on the etiology of DNIs.

According to our epidemiology, we propose as empirical therapy the intravenous use of a beta-lactam /beta-lactamase inhibitor; metronidazole or clindamycin can be added only in specific cases such as in immunocompromised patients. Although clindamycin has a broad range of activity against aerobic Gram-positive cocci and anaerobic organisms, clinicians must be prudent with its usage due to the increased rate of resistance. On the other hand, metronidazole has excellent in vitro activity against most obligate anaerobic bacteria (*Bacteroides, Fusobacterium,* etc*)* but is inactive for *Propionibacterium acnes* and *Actinomyces israelii.*

In the present study, cultures have obtained from 462 patients; 252 were unimicrobial, 3 were polymicrobial and 207 were culture-negative. The microorganisms isolated were bacteria that were part of the pharyngeal flora such as *S. pyogenes S. aureus, Streptococcus group C, Streptococcus anginosus, Fusobacterium* spp. and *Prevotella* spp*.* Previous studies have also demonstrated that *S. pyogenes, S. aureus, Streptococcus viridans* and *Haemophilus influenza* are the most common bacterial species [22–25]. The most common bacteria isolated were *S. pyogenes* and *S. aureus* in adults and in children as well. Most studies report a lower prevalence of DNI in children compared to adults [3, 26, 27]. Probably this may be caused by the history of antibiotics abuse, especially in colds and other viral infections, which are more prevalent in children than in adults [28, 29]. In our study children comprised 5,6% of total patients. According to the literature, the effect of age on the distribution of bacteria causing DNI is not clear. Coticchia et al. have compared the bacteriology between patients less than 1 year and above 1 year of age and they have found that age was a significant factor influencing bacteriology of DNI [30]. On the other hand, other authors did not find any significant differences in bacteriology of DNI between various age groups. We point out that the incidence of anaerobic bacteria was higher in adults compared to children.

Finally, interesting finding was that 16S rRNA PCR followed by sequencing analysis detected bacterial DNA in 33 specimens that gave culture-negative results; 19 of them were found to be positive for anaerobic bacteria such as *A. israelii* and *F. necrophorum*. Since none of these patients had taken antimicrobial therapy before admission, the failure of the conventional cultures to isolate these microorganisms could be related to the inappropriate sample collection combined with the fragility of the bacteria and the short incubation time of the anaerobic culture. In addition, 16S rRNA PCR identified correctly the causative microorganisms which were isolated from 150 samples, while, the results obtained by the molecular methods were available sooner than that obtained from cultures (mean time 2 versus 5 days). However, this molecular assay failed to detect bacterial DNA in 105 culture-positive samples, probably due to the low microbial load. It is known that the sensitivity of the 16S rRNA PCR, when it is applied directly to the clinical samples, is depending on the bacterial concentration. The culture of the low-microbial load clinical samples combined with an elongation of the time of incubation time enhances the growth of microorganisms, giving more positive results than the molecular method. Unfortunately, this molecular approach, which uses the 16S rRNA PCR combined with Sanger analysis, is not able to identify more than one microorganism per sample. Probably in the future, the implementation of the next generation sequencing technology could solve this limitation.

## Conclusions

In conclusion, deep neck infections represent a medical and surgical emergency, they are still common and can develop serious complications. ‘Evidence-guided’ empirical treatment with IV antibiotics at the time of diagnosis is mandatory, highlighting the importance of epidemiological studies in DNIs microbiology. Although, in our study, the main pathogens were *S. pyogenes* and *S. aureus,* the combination of conventional and molecular assays revealed that anaerobic bacteria play also a significant role in the pathogenesis of DNIs. Based on the local epidemiology, we propose as empirical therapy the intravenous use of a beta-lactam /beta-lactamase inhibitor; metronidazole or clindamycin can be added only in specific cases such as in immunocompromised patients.

## Data Availability

The datasets are available by request to the corresponding author.

## Abbreviations

DNIs: Deep neck infections
EUCAST: European Committee on Antimicrobial Susceptibility Testing
PCR: Polymerase chain reaction
